# Fluorescent Marker Monitoring and Nurse-Led Feedback for Operating Room Environmental Cleaning: A Quasi-Experimental Single-Group Pre-Post Study

**DOI:** 10.3390/nursrep16080261

**Published:** 2026-07-29

**Authors:** Paulo Brois, Sofia Rita, Marco Serafim, Soraia Pereira, Fernanda Príncipe, Henrique Pereira, Liliana Mota

**Affiliations:** 1 Clean Hospitals, 1262 Eysins, Switzerland; paulo.brois@cleanhospitals.com; 2 Jean Piaget Algarve School of Health, Piaget Institute, 8300-025 Silves, Portugal; 3 Baixo Alentejo Local Health Unit, 7801-849 Beja, Portugal; sofia.rita@ulsba.min-saude.pt; 4 Kingston Hospital NHS Foundation Trust, Kingston-upon-Thames, London KT2 7QB, UK; marco.serafim@nhs.net; 5 Nursing School, University of Porto, Rua Dr. António Bernardino de Almeida 830/844/856, 4200-072 Porto, Portugal; soraiapereira@esep.up.pt; 6 RISE-Health, Nursing School, University of Porto, Rua Dr. António Bernardino de Almeida 830/844/856, 4200-072 Porto, Portugal; 7 RISE-Health, Faculty of Medicine, University of Porto, 4200-319 Porto, Portugal; vice.presidente@essnortecvp.pt; 8 Northern Health School of the Portuguese Red Cross, 3720-126 Oliveira de Azeméis, Portugal; direcao@essnortecvp.pt

**Keywords:** operating rooms, environmental monitoring, infection control, perioperative nursing, quasi-experimental study, quality improvement, patient safety

## Abstract

**Background:** Environmental hygiene in operating rooms is essential for infection prevention, but visual inspection alone may not reliably identify missed cleaning. **Aim:** To assess the feasibility of fluorescent gel marker monitoring with ultraviolet light, combined with a nurse-led educational and feedback intervention, on the cleaning of high-touch operating room surfaces. **Methods:** A quasi-experimental, nonrandomized, single-group pre-post study was conducted in five operating rooms of a district general hospital in southern Portugal. Forty-two cleaning episodes and 420 surface assessments were evaluated across three sequential phases: blinded baseline monitoring, post-training monitoring after a 45-min nurse-led workshop, and feedback-based monitoring using an ultraviolet walk-through. The primary outcome was the proportion of surface assessments classified as completely clean. Exploratory Mann–Whitney U tests compared ordinal cleaning outcomes between phases; *p*-values were nominal and not adjusted for multiple comparisons. **Results:** Complete cleanliness increased from 44.7% at baseline to 69.3% after training and 89.2% during feedback-based monitoring. The largest observed increases occurred among surfaces with poor baseline performance, particularly anesthesia machine rebreathing bags, IV stands, computer keyboards, and computer mice. Visual inspection identified dust on keyboards and occasional medication residue but missed several surfaces detected by fluorescent marker assessment. **Conclusions:** Fluorescent marker monitoring with ultraviolet feedback was feasible within the study setting and was accompanied by progressive improvements in the proportion of surfaces classified as completely clean. However, the absence of a concurrent control group precludes attributing these changes exclusively to the intervention. The approach may support nurse-led quality improvement in operating room environmental hygiene.

## 1. Introduction

Healthcare-associated infections (HAIs) remain a major threat to patient safety. They are associated with increased morbidity and mortality, length of hospital stay, antimicrobial resistance and healthcare costs [1]. Environmental hygiene is one component of multimodal infection prevention.

Research has shown that environmental hygiene interventions are associated with reductions in environmental bioburden and, in some settings, patient colonization or HAIs [2]. More recently, a stepped-wedge cluster-randomized trial reported that enhanced cleaning and disinfection of shared medical equipment was associated with a relative reduction in HAIs of 34.5% and an absolute reduction of 5.2% [3].

The operating room (OR) is a highly controlled, high-risk clinical environment. Temperature, humidity, air exchange, traffic control, aseptic practice, and environmental cleaning all contribute to maintaining a safe surgical environment [4,5,6]. High-touch surfaces and reusable noncritical equipment may become contaminated during patient care and may contribute to indirect transmission via healthcare workers’ hands, shared equipment, or the surrounding patient environment [5,6,7]. International environmental cleaning guidance therefore emphasizes risk-based procedures, clearly assigned responsibilities, training, checklists, and routine monitoring with feedback [7].

OR efficiency is commonly assessed using performance indicators such as turnover time [8]. Shorter turnover times may improve access to surgical care and optimize resource use [9,10], but it may also compress the time available for environmental cleaning and disinfection. Previous studies have shown that OR cleaning may be insufficient or inconsistent with expected standards, particularly for high-touch surfaces and shared equipment [11,12]. In busy perioperative workflows, time pressure may further compromise cleaning consistency.

For this reason, environmental cleaning should not be assessed solely by whether the room appears visually clean after turnover. Given the importance of environmental control and adequate decontamination of OR surfaces, monitoring environmental cleaning is essential to determine whether it has been performed effectively. Several methods have been described for evaluating environmental cleaning, including visual inspection, microbiological methods, adenosine triphosphate (ATP) bioluminescence, and fluorescent markers combined with ultraviolet (UV) light [13].

A broader infection prevention perspective is also important. Intraoperative traffic, door openings, and staff behaviour can affect airflow, microbial air contamination, concentration of personnel, and adherence to infection prevention practices [14,15,16]. Recent work on OR door openings suggests that the clinical effect on surgical site infection risk is probably modest and that the certainty of evidence remains very low, but cumulative risk may be more relevant for patients with higher baseline surgical site infection risk [15]. Automated traffic monitoring has also shown that OR traffic can be higher than staff expect, highlighting the potential value of objective measurement and feedback in complex perioperative environments [17]. Although traffic monitoring and surface cleaning assessment measure different processes, both reflect the same principle: subjective impressions of safe practice may not be sufficient, and objective feedback can support improvement.

Perioperative nurses are well positioned to coordinate environmental hygiene improvement because their practice combines patient safety, infection prevention, workflow coordination, team education, and quality assurance [4]. This role is consistent with evidence on infection control link nurses, who have been described as clinical role models trained to monitor infection prevention-related issues, inform and facilitate peers, and support improvements in everyday clinical practice [18]. In this context, nurse-led environmental hygiene monitoring can provide a practical bridge between infection prevention standards and the realities of perioperative workflow, particularly when it combines objective assessment methods, structured feedback, staff engagement, and adaptation to routine practice.

Within this workflow, rapid turnover is an important performance indicator [8], but it may create tension between operational efficiency and rigorous cleaning standards [11]. This study addresses the gap between perceived and actual cleanliness by positioning objective monitoring as a practical tool for nursing-led quality assurance [19]. Therefore, this study aimed to assess the feasibility and effect of fluorescent gel marker monitoring with ultraviolet light, combined with a nurse-led educational and feedback intervention, on the cleaning of high-touch OR surfaces. Given the nonrandomized single-group design, the study was intended to describe between-phase changes and generate preliminary evidence rather than establish a causal intervention effect. This approach may contribute to the ongoing improvement of environmental cleaning protocols, supporting efforts to reduce the risk of transmission of HAIs during surgery and to improve quality of care and patient safety.

## 2. Materials and Methods

**Figure 1:**
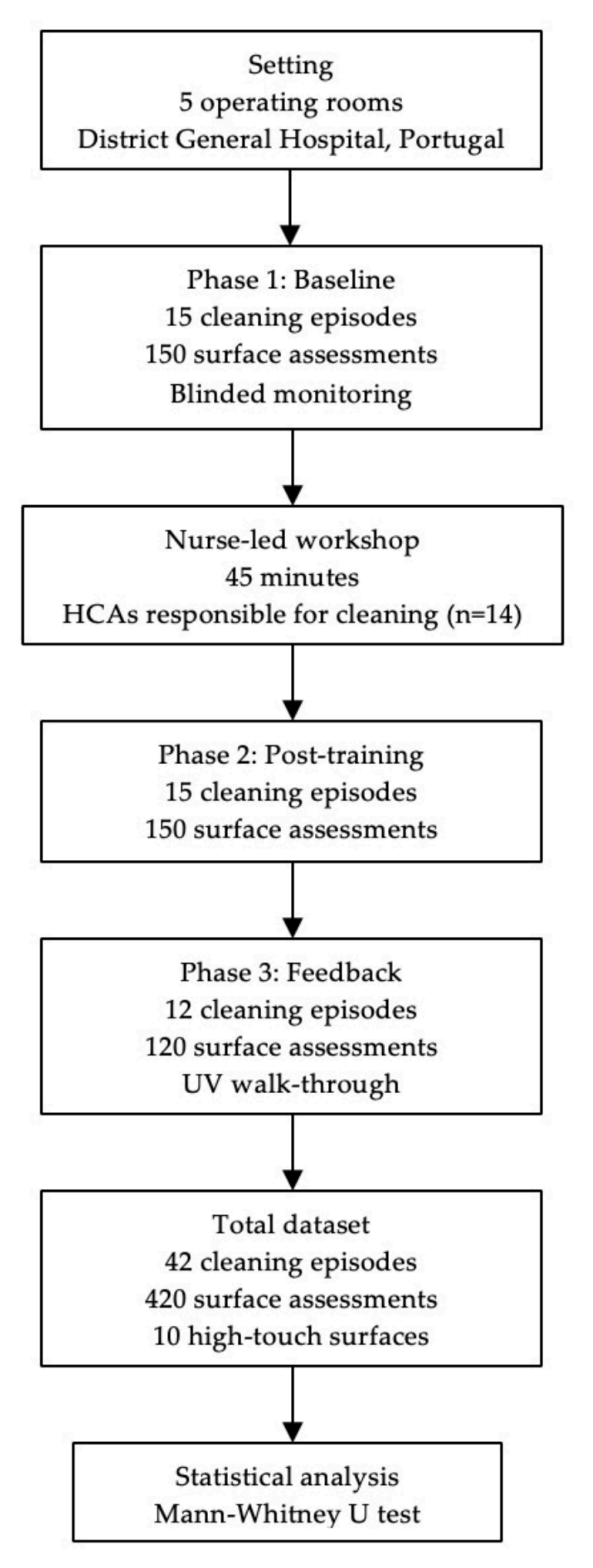
TREND flow diagram of cleaning episodes and surface assessments across the three study phases.

This quasi-experimental, nonrandomized, single-group pre-post intervention study was conducted in accordance with the Transparent Reporting of Evaluations with Nonrandomized Designs (TREND) statement. The study was structured into three sequential phases over a 2-month period: (1) blinded baseline monitoring of cleaning effectiveness; (2) a nurse-led educational intervention followed by post-training monitoring; and (3) feedback-based monitoring in which HCAs viewed residual fluorescent markers with the researcher using UV light immediately after cleaning (Figure 1). No concurrent control group was included. Therefore, observed differences between phases were interpreted as temporal changes associated with the implementation of the intervention and not as definitive evidence of causality.

The study was conducted in all five ORs of the surgical department of a district general hospital in southern Portugal. These rooms were used for adult surgical activity across several specialties, including orthopedics, general surgery, gynecology, obstetrics, and urology. The ORs supported elective and urgent surgery and therefore reflected routine variability in perioperative workflow, turnover pressure, and staffing.

The socio-demographic characteristics of the HCAs responsible for OR cleaning are presented in Table 1. No HCAs were excluded after enrolment, and no losses to follow-up occurred during the intervention and evaluation phases.

The study used the following operational definitions: a cleaning episode was one complete cleaning process performed after a surgical procedure or at the end of a surgical program; a surface assessment was the evaluation of one predefined high-touch surface within a cleaning episode; and a fluorescent marker evaluation was the UV-light assessment of one or two marker applications on a surface. The primary analysis was conducted at the surface-assessment level.

Ten high-touch surfaces were selected according to perioperative environmental cleaning guidance and clinical relevance to infection prevention [6,20]. The selected surfaces included equipment such as IV stands, surgical lights, and anesthesia machine rebreathing bags, which are often overlooked during rapid turnover transitions [8]. A total of 420 surface assessments were performed across the predefined high-touch surfaces between April and May 2022. These assessments were conducted across three sequential study phases: 150 surface assessments at baseline, before the educational intervention; 150 surface assessments after the educational intervention; and 120 surface assessments during the third-week feedback-monitoring phase. No surface assessments were performed after surgical procedures involving patients known to be infected or colonized with multidrug-resistant organisms.

All surface assessments were conducted under routine clinical conditions by a single researcher.

For each cleaning episode, contextual data were also collected from patient records and perioperative documentation related to the corresponding surgical procedure. Patient-identifiable information was not included in the research dataset. Patient- and procedure-related variables included surgical specialty, originating department, documented infection or colonization with multidrug-resistant organisms, antibiotic therapy and/or prophylaxis before surgery, and wound classification according to the Altemeier Classification (clean, clean-contaminated, contaminated, or dirty/infected) [21]. Additional workflow- and cleaning-related variables included surgical urgency classification (elective, urgent, or emergency), type of anesthesia administered (local, intravenous general, balanced general, epidural, subarachnoid block, sequential block, or nerve plexus block), cleaning products used for environmental disinfection, number of HCAs involved in the cleaning process, turnover time, duration of cleaning activities, occurrence of room change between surgeries within the same surgical program, and the total number of surgeries scheduled in that program.

Surface cleanliness was assessed using two methods. First, visual inspection was performed to identify visible blood, wound exudate, organic fluids, saline crystals, creams or ointments, oils, solutes, dyes, dust, medication residue, or other visible soil. Second, a commercially available fluorescent gel marker visible under UV light was used to assess whether the surface had been cleaned.

Fluorescent gel markers were applied to the ten predefined high-touch surfaces selected for cleaning assessment (Table 2) by a single member of the research team at the end of surgery and before HCAs entered the OR to clean. For smaller surfaces (<50 cm), including the anesthesia machine rebreathing bag, keyboard, and computer mouse, one mark of approximately 1 cm in diameter was applied to a randomly selected area and spread in a circular motion until it was no longer visible under normal lighting. For larger surfaces (>50 cm), including the operating room table, instrument table, IV stand, surgical lights, anesthesia machine table, anesthesia cart, and garbage bin, two applications with the same characteristics were placed on randomly selected areas.

After cleaning, the researcher assessed marker removal using the same UV light source throughout the study. Surface cleanliness was classified using a standardized three-level system adapted from Hung et al. [22]: completely clean if less than 25% of the fluorescent residue remained, partially cleaned if 25% to 75% remained, and not cleaned if more than 75% remained visible under UV light. When two marks were applied to the same surface, the average percentage of residual fluorescent marker was used to classify the surface assessment. To ensure consistency and standardization, all marker applications and UV-light assessments were performed by a single trained researcher using the same UV light source throughout the study.

The educational intervention consisted of a 45-min workshop led by a perioperative nurse researcher. The learning objectives were to: (1) reinforce the role of environmental cleaning in breaking the chain of infection; (2) discuss limitations of visual inspection; (3) review local OR cleaning and disinfection procedures; (4) identify high-touch surfaces commonly missed during turnover; and (5) introduce fluorescent marker monitoring as an educational and quality improvement tool [6]. Teaching strategies included a brief presentation, discussion of baseline environmental hygiene principles, demonstration of fluorescent marker visibility under UV light, and practical reflection on the OR workflow.

During the feedback phase, HCAs accompanied the researcher during point-by-point UV assessment after cleaning. Residual fluorescent marks were shown immediately, and surfaces classified as partially cleaned or not cleaned were cleaned again as part of the usual workflow. Feedback was delivered in a constructive, non-punitive manner and focused on the surface and process rather than individual blame. This walk-through strategy was intended to integrate audit, immediate feedback, and experiential learning into routine OR turnover.

The primary outcome was the proportion of surface assessments classified as completely clean. Secondary outcomes were the distribution of surface assessments across the three ordinal categories (not cleaned, partially cleaned, completely clean), differences between study phases for each monitored surface, and descriptive contextual variables related to cleaning episodes and workflow.

Statistical analysis was performed using SPSS (v.24). Categorical variables were reported as counts and percentages, and continuous variables were reported as means and standard deviations. Cleaning outcome was coded as an ordinal variable with three ordered categories: not cleaned, partially cleaned, and completely clean. Given the ordinal nature of this outcome, between-phase comparisons were performed using the Mann–Whitney U test. The main comparisons were baseline versus post-training and baseline versus third-week feedback-monitoring assessments. These comparisons were intended to estimate the direction and magnitude of observed between-phase differences. They were not interpreted as estimates of a causal intervention effect because the study lacked randomization and a concurrent control group. Post-training versus feedback-monitoring comparisons, when reported, were considered exploratory.

Because this was an exploratory, single-centre study with a limited number of assessments per surface, formal adjustment for multiple comparisons was not applied. Statistical significance was set at *p* < 0.05. Accordingly, the analyses should be considered exploratory and hypothesis-generating rather than confirmatory. Nominal *p*-values were interpreted alongside the direction, magnitude, and clinical relevance of the observed differences. This study was conducted as a quality improvement and infection prevention initiative using a quasi-experimental nonrandomized, single-group pre-post design. As the study did not involve randomization, allocation of participants to intervention groups, therapeutic interventions, or evaluation of clinical treatment outcomes, it was not considered a clinical trial requiring prospective registration in a public trial registry. Ethical approval was obtained prior to study commencement (EDOC/2022/9691). The proposed research study did not entail any risks or costs for the participants. No patient-identifiable information was included in the research dataset; procedural data extracted from surgical records were anonymized and used only to characterize cleaning episodes. Data were coded, stored securely, and analyzed without identifying individual patients, HCAs, or surgical teams.

## 3. Results

The study included 42 cleaning episodes and 420 surface assessments across the three study phases: 15 cleaning episodes and 150 surface assessments at baseline, 15 cleaning episodes and 150 surface assessments after training, and 12 cleaning episodes and 120 surface assessments during the feedback-monitoring phase.

### 3.1. Descriptive Analysis of Baseline Cleaning Episodes

During the baseline phase, 15 cleaning episodes were assessed; 12 (80.0%) occurred between surgeries and three (20.0%) occurred after the end of the surgical program. Of these 15 cleaning episodes, seven (46.7%) were conducted after orthopedic surgeries, five (33.3%) after general surgery procedures, one (6.7%) after gynecological surgery, one (6.7%) after obstetric surgery, and one (6.7%) after urological surgery.

Seven (46.7%) patients came from the orthopedic department, three (20%) from the general surgery department, two (13.3%) from the Ambulatory Surgery Unit (ASU), one (6.7%) from gynecology, one (6.7%) from obstetrics, and one (6.7%) from urology.

All cleaning episodes (100.0%) followed surgical procedures in which antibiotic prophylaxis with 2 g of cefazolin was administered according to the departmental protocol. Regarding the classification of the surgical wound, twelve (80%) were classified as clean and three (20%) as clean-contaminated.

Regarding the classification of surgeries by urgency, nine (60%) were elective surgeries and six (40%) were urgent surgeries. The predominant type of anesthesia was spinal anesthesia performed in eight (53.3%) patients, followed by balanced general anesthesia performed in four (26.7%) patients, sequential block in two (13.3%) patients, and intravenous general anesthesia in one patient (6.7%).

The number of HCAs involved in cleaning and disinfection was one in four cleaning episodes (26.7%) and two in eleven cleaning episodes (73.3%). The presence of only one HCA in the cleaning resulted from sudden absences from work by these professionals due to health issues, which posed constraints on their replacement.

The number of scheduled surgeries was recorded only for elective surgery programs; six cleaning episodes occurred in programs with two scheduled surgeries (40.0%), and three cleaning episodes occurred in programs with three scheduled surgeries (20.0%). The remaining six cleaning episodes (40.0%) followed urgent surgeries, for which there was no scheduled surgical program.

The time for cleaning and disinfection of the ORs varied between 14 min and 45 min, with an average of 24.93 min (SD = 8.98), as well as the turnover time, which ranged from 22 min to 50 min, with an average time of 35.14 min (SD = 9.39). The longer periods were influenced by the reduced number of HCAs (two members for five rooms) associated with more than two rooms finishing simultaneously.

No room changes occurred in any cleaning episode during cleaning and disinfection between surgeries. No cleaning episode followed a surgical procedure involving a patient under contact isolation.

Regarding data collection through visual inspection of surfaces before applying the fluorescent gel markers, dust was observed in all 15 keyboard surface assessments before fluorescent marker application (100%), and white medication residue was found in two anesthesia cart surface assessments (13.3%). No residue or dirt was detected on the remaining surfaces (0%).

### 3.2. Descriptive Analysis of Post-Training Cleaning Episodes

Data collection was conducted after the training session and with the knowledge of the multidisciplinary team. A total of 15 cleaning episodes were assessed; 12 (80.0%) occurred between surgeries and three (20.0%) occurred after the end of the surgical program. Of these 15 cleaning episodes, six (40.0%) followed orthopedic surgeries, six (40.0%) followed general surgery procedures, two (13.3%) followed gynecological surgery, and one (6.7%) followed urological surgery.

Five patients (46.7%) came from the orthopedic department, five (20%) from the general surgery department, four (13.3%) from the Ambulatory Surgery Unit (ASU), and one (6.7%) from gynecology.

In this second phase, eleven cleaning episodes (73.3%) followed surgical procedures in which antibiotic prophylaxis with 2 g of cefazolin was administered, whereas four cleaning episodes (26.7%) followed procedures for which antibiotic prophylaxis was not indicated. Regarding the classification of the surgical wound, eleven (73.3%) were classified as clean, one (6.7%) as clean-contaminated, and three (20%) as contaminated.

All cleaning episodes (100.0%) followed elective surgical procedures.

Eight cleaning episodes (53.3%) followed surgeries performed under balanced general anesthesia, six (40.0%) followed surgeries performed under spinal anesthesia, and one (6.7%) followed surgery performed under local anesthesia.

The number of HCAs involved in cleaning and disinfection was one in two cleaning episodes (13.3%), two in ten cleaning episodes (66.7%), and three in three cleaning episodes (20.0%).

Regarding the number of scheduled surgeries, six cleaning episodes (40.0%) occurred in ORs with three scheduled surgeries, eight cleaning episodes (53.3%) occurred in ORs with two scheduled surgeries, and one cleaning episode (6.7%) occurred in an OR with one scheduled surgery.

The time for cleaning and disinfection of the ORs varied between 14 min and 28 min, with an average of 19.20 min (SD = 3.34), and the turnover time ranged from 19 min to 32 min, with an average time of 24.50 min (SD = 3.58).

No room change occurred in any cleaning episode. No cleaning episode followed a surgical procedure involving a patient under contact isolation.

Dust was still observed among the keys in all 15 keyboard surface assessments (100%) before fluorescent marker application. No residue or dirt was detected on the remaining surfaces (0%).

### 3.3. Descriptive Analysis of Feedback-Monitoring Cleaning Episodes

Data collection was conducted with each HCA accompanying the point-to-point monitoring, proceeding with the respective cleaning in surfaces that were not completely clean. A total of 12 cleaning episodes were assessed, all of which occurred between surgeries (100.0%). Of these 12 cleaning episodes, nine (75.0%) followed orthopedic surgeries and three (25.0%) followed general surgery procedures.

Nine patients (75%) came from the orthopedic department, and three (25%) from the general surgery department.

Eleven patients (91.7%) received antibiotic prophylaxis, while one (8.3%) did not have an indication. Regarding the classification of the surgical wound, eleven (91.7%) were classified as clean, and one (8.3%) was classified as clean-contaminated. Regarding surgical urgency, eight cleaning episodes (66.7%) followed urgent surgeries and four (33.3%) followed elective surgeries.

The predominant type of anesthesia was spinal anesthesia performed in nine patients (75%), followed by balanced general anesthesia performed in two patients (16.7%), and brachial plexus block in one patient (8.3%).

The number of HCAs involved in cleaning and disinfection was two in eleven cleaning episodes (91.7%) and one in one cleaning episode (8.3%).

The number of scheduled surgeries was recorded only for elective surgery programs; two cleaning episodes (16.7%) occurred in programs with two scheduled surgeries, and two cleaning episodes (16.7%) occurred in programs with three scheduled surgeries. The remaining eight cleaning episodes (66.7%) followed urgent surgeries, for which there was no scheduled surgical program.

The time for cleaning and disinfection of the ORs varied between eighteen minutes and 35 min, with an average of 23.75 min (SD = 5.97), and the turnover ranged from 21 min to 32 min, with an average of 25.75 min (SD = 5.19).

No room change occurred in any cleaning episode. No cleaning episode was assessed at the end of a surgical program or after a surgical procedure involving a patient under contact isolation. In the data collection conducted through visual inspection of surfaces, dust was still observed among the keys of all 12 keyboards (100%) assessed before fluorescent marker application. No residue or dirt was detected on the remaining surfaces (0%).

### 3.4. Observed Differences in Cleaning Outcomes Across Study Phases

As shown in Table 3, the operating room table was consistently classified as completely clean across all three study phases. In contrast, the anesthesia machine rebreathing bag was not cleaned at baseline, with improvement observed after training and during feedback-based monitoring. The remaining surfaces also showed progressive improvement across the three study phases.

Overall, the observed proportion of surface assessments classified as completely clean increased from 44.7% at baseline to 69.3% after training and 89.2% during feedback-based monitoring, corresponding to absolute differences from baseline of 24.7 and 44.5 percentage points, respectively. Exploratory Mann–Whitney U comparisons between baseline and post-training assessments suggested improvements in IV stands (U = 170.0; *p* = 0.016), surgical lights (U = 175.5; *p* = 0.008), and anesthesia machine rebreathing bags (U = 180.0; *p* = 0.004). Surfaces associated with clinical documentation, such as keyboards (*p* = 0.015) and computer mice (*p* = 0.029), also showed marked improvement, which is relevant because these high-touch surfaces may contribute to indirect transmission when cleaning is inconsistent. Because multiple surface-level comparisons were performed and no formal adjustment for multiple comparisons was applied, these *p*-values are reported as nominal and should be interpreted cautiously.

Exploratory analyses of contextual procedural variables did not identify statistically significant associations with surface cleanliness outcomes. These differences should not be interpreted as causal effects of the intervention because concurrent temporal, organizational, staffing, and observer-related factors could not be excluded.

## 4. Discussion

To improve and maintain a clean and safe environment in ORs, it is necessary to use objective methods that allow cleaning practices to be assessed in a simple, feasible, and timely manner. Such methods should provide immediate information, support corrective measures, and be useful as educational tools. The Joint Commission International emphasizes fluorescent marking not only as a method for evaluating environmental cleaning, but also as a strategy for the ongoing education of cleaning teams [23].

In this study, fluorescent gel marker monitoring with UV light identified incomplete cleaning of several high-touch surfaces that appeared visually clean. This finding supports the view that visual inspection alone is insufficient to assess the effectiveness of environmental cleaning. Although visual inspection detected dust on keyboards and occasional medication residue on the anesthesia cart, fluorescent marker assessment provided more detailed information on whether surfaces had been subjected to adequate mechanical cleaning action. These findings are consistent with previous reports highlighting the limitations of visual inspection and the value of objective environmental monitoring methods [7,24].

The presence of dust on keyboards was observed at every stage of the study. This finding is relevant because keyboards and computer mice are frequently touched during perioperative workflow and may be difficult to clean effectively, particularly when they are not designed for healthcare environments. The use of washable keyboards and computer mice suitable for clinical settings could therefore contribute to improved environmental hygiene. Medication residue observed on the anesthesia cart during the baseline phase also suggests that some surfaces may not have been systematically inspected or cleaned before the educational intervention.

The relevance of anesthesia-related surfaces should also be emphasized. In the baseline phase, the anesthesia machine rebreathing bag was not completely cleaned in any assessment, but substantial improvement was observed after training and during feedback-based monitoring. Previous outbreak reports have described perioperative infection risks related to contaminated medications, including propofol contamination with Serratia marcescens, and have highlighted contributing factors such as breaches in aseptic technique, unsafe medication handling, and time pressure during rapid turnover [25]. Although the present study did not assess microbiological contamination or medication-handling practices, these reports illustrate how perioperative workflow pressures may contribute to infection prevention vulnerabilities.

The improvement observed after the educational intervention suggests that structured training may have contributed to better recognition of high-touch surfaces and greater awareness of the limitations of visual inspection. In this study, exploratory differences between baseline and post-training assessments were observed for IV stands, surgical lights, anesthesia machine rebreathing bags, computer keyboards, and computer mice. However, because multiple surface-level comparisons were performed and no formal adjustment for multiple comparisons was applied, these *p*-values should be interpreted as nominal and exploratory. The emphasis should therefore be placed not only on statistical significance, but also on the direction and magnitude of improvement across phases.

These results are consistent with the REACH trial, a pragmatic, multicentre, stepped-wedge randomised trial in which a multimodal environmental cleaning bundle was implemented in 11 Australian hospitals [26]. The bundle focused on optimising product use, cleaning technique, staff training, auditing with feedback, and communication for routine cleaning. Cleaning thoroughness of frequent touch points was assessed using fluorescent marking gel and improved substantially during the intervention. Although the REACH trial was conducted in broader acute-care hospital settings rather than specifically in ORs, its findings support the use of structured education, clearly defined cleaning processes, identification of frequent-touch surfaces, audit, and feedback as key components of environmental cleaning improvement programmes [26].

In the present study, the training session was designed to be constructive and non-punitive. The role of HCAs in infection prevention and control was emphasized, and the updated environmental decontamination standard for ORs was reviewed. Importantly, the results of the baseline assessment were not disclosed before the post-training data collection. This approach allowed the intervention to focus on improvement and professional engagement rather than on individual blame. Presenting only the baseline results to the whole team could have been perceived as intimidating and might have reduced the constructive value of the intervention.

A possible Hawthorne effect should also be considered. After the baseline phase, HCAs and the wider perioperative team were aware that OR cleaning effectiveness was being monitored, which may have influenced cleaning behaviour. This observer effect is frequently discussed in infection prevention research and may contribute to behaviour change when healthcare workers know that their practice is being observed [27]. In this study, it is unlikely that the Hawthorne effect influenced the baseline phase, because monitoring was blinded at that stage. However, it may have contributed to the improvements observed after training and during feedback-based monitoring.

Rather than interpreting the Hawthorne effect only as a limitation, it can also be considered as one possible mechanism through which audit and feedback interventions influence practice. Visible monitoring, clinical supervision, and immediate feedback may reinforce attention to high-touch surfaces and promote adherence to cleaning standards [11,28,29]. Nevertheless, this effect may decrease over time, and the short follow-up period of the present study does not allow conclusions about the sustainability of the observed improvement [28]. Strategies such as longer follow-up, intermittent monitoring, pauses between data collection periods, and unannounced assessments may help reduce or better understand this effect in future studies [29].

During the final phase, HCAs accompanied the researcher during UV-light assessment and were able to visualize residual fluorescent markers immediately after cleaning. This walk-through feedback strategy allowed missed surfaces to be identified and cleaned again as part of the usual workflow. Although the improvement from post-training to feedback-based monitoring was mainly descriptive and should be interpreted cautiously, this phase appeared useful as an experiential learning process. By making missed areas visible, the intervention helped translate cleaning standards into concrete, surface-specific feedback.

The possible influence of visual feedback on attention and memory retention is an interesting area for future research. Previous research suggests that color can influence attention and memory performance, and that color information may support visual encoding and recognition memory. In the present study, however, this mechanism was not directly assessed; therefore, the role of fluorescent color and UV visualization should be interpreted only as a hypothesis for future research [30,31].

This study has limitations. The sample size was small and was limited by the available study period, requiring caution when interpreting and generalizing the results. The study was conducted in a single centre and did not include a control group, which limits causal inference. The repeated assessment of surfaces within the same ORs, cleaning episodes, surface types, and potentially the same HCAs may have introduced clustering, which was not accounted for in the statistical analysis. In addition, all fluorescent marker applications and UV-light assessments were performed by a single researcher, which supported consistency but may have introduced observer bias. The study also did not include microbiological or ATP assessment; therefore, fluorescent marker removal should be interpreted as evidence of mechanical cleaning action rather than direct evidence of microbial reduction.

There is also a possibility that the Hawthorne effect influenced the observed behavioural changes. Future studies should include larger randomly selected samples, longer follow-up, repeated or unannounced monitoring, and, where possible, more than one assessor. Complementary microbiological or ATP assessment would also strengthen interpretation of the relationship between fluorescent marker removal, environmental bioburden, and infection prevention outcomes.

Despite these limitations, this study suggests that fluorescent gel marker monitoring with UV feedback is feasible in routine OR practice and may support nurse-led quality improvement in perioperative environmental hygiene. The approach provided immediate, visible, and actionable feedback, helped identify high-touch surfaces that were frequently missed, and may contribute to the ongoing improvement of cleaning and disinfection protocols in ORs.

## 5. Conclusions

The findings of this study indicate that visual inspection alone may be insufficient to reliably assess the effectiveness of operating room surface cleaning. Practical and organizational factors, including training needs, supervision, time constraints, and workflow pressures, may influence cleaning performance and contribute to inconsistent outcomes.

Fluorescent gel marker monitoring with ultraviolet light was feasible within the study setting and provided an objective means of identifying high-touch surfaces that had not been adequately cleaned despite appearing visually clean. More favourable cleaning outcomes were observed after the educational intervention and during feedback-based monitoring, particularly for IV stands, surgical lights, anesthesia machine rebreathing bags, computer keyboards, and computer mice. However, because this was a nonrandomized single-group study without a concurrent control group, these changes cannot be attributed exclusively to the intervention.

The integration of fluorescent marker monitoring into routine infection prevention practice may contribute to more structured audit, timely feedback, immediate corrective action, and greater staff engagement in environmental hygiene. As part of a nurse-led quality improvement strategy, this approach may help reinforce continuous learning, accountability, and a consistent culture of safety in perioperative settings.

These findings should be regarded as preliminary and hypothesis-generating. Further multicentre controlled studies with larger samples, longer follow-up, appropriate consideration of clustering, and complementary microbiological or ATP assessment are needed to confirm the effectiveness, sustainability, and clinical relevance of this intervention.

## Figures and Tables

**Table 1:** Socio-demographic characteristics of HCAs (n = 14).

Characteristics	N	%
Age (mean, SD)	43.93 (SD = 10.06)	
Gender		
Male	1	7.14
Female	13	92.86
Qualifications		
Basic education	9	64.29
Secondary education	5	35.71
Time of professional practice in the institution, in years (mean, SD)	9.29 (SD = 8.47)	
Time of professional practice in the OR department, in years (mean, SD)	5.57 (SD = 6.02)	

**Table 2:** High-touch surfaces.

High-Touch Surface	Length (cm)	Number of Marks
Operating room table	>50	2
Instrument table	>50	2
IV stand	>50	2
Surgical lights	>50	2
Anesthesia machine table	>50	2
Anesthesia machine rebreathing bag	<50	1
Anesthesia cart	>50	2
Computer keyboard	<50	1
Computer mouse	<50	1
Operating room garbage bin	>50	2

**Table 3:** Comparative Analysis of Surgical Surface Cleaning Evaluation Across Study Phases Using Fluorescent Gel Marker Assessment.

High-Touch Surfaces	Level of Cleanliness	Baseline		Post-Training		Feedback Phase	
		%	n	%	n	%	n
Operating room table	Not cleaned	0	0	0	0	0	0
	Partially cleaned	0	0	0	0	0	0
	Completely clean	100	15	100	15	100	12
Instrument table	Not cleaned	0	0	0	0	0	0
	Partially cleaned	13.3	2	6.7	1	0	0
	Completely clean	86.7	13	93.3	14	100	12
IV stand	Not cleaned	73.3	11	26.7	4	16.7	2
	Partially cleaned	13.3	2	20	3	8.3	1
	Completely clean	13.3	2	53.3	8	75	9
Surgical lights	Not cleaned	60	9	6.7	1	0	0
	Partially cleaned	20	3	40	6	33.3	4
	Completely clean	20	3	53.3	8	66.7	8
Anesthesia machine table	Not cleaned	26.7	4	13.3	2	8.3	1
	Partially cleaned	0	0	6.7	1	0	0
	Completely clean	73.3	11	80	12	91.7	11
Anesthesia machine rebreathing bag	Not cleaned	100	15	40	6	0	0
	Partially cleaned	0	0	0	0	8.3	1
	Completely clean	0	0	60	9	91.7	11
Anesthesia cart	Not cleaned	40	6	26.7	4	0	0
	Partially cleaned	6.7	1	0	0	8.3	1
	Completely clean	53.3	8	73.3	11	91.7	11
Computer keyboard	Not cleaned	93.3	14	40	6	0	0
	Partially cleaned	0	0	20	3	16.7	2
	Completely clean	6.7	1	40	6	83.3	10
Computer mouse	Not cleaned	86.7	13	40	6	0	0
	Partially cleaned	0	0	0	0	0	0
	Completely clean	13.3	2	60	9	100	12
Operating room garbage bin	Not cleaned	20	3	0	0	0	0
	Partially cleaned	0	0	20	3	8.3	1
	Completely clean	80	12	80	12	91.7	11

## Data Availability

The datasets generated and analysed during the current study are available from the corresponding author upon reasonable request. The data are not publicly available due to ethical and confidentiality restrictions; however, access to anonymized data may be granted upon request.

## Public Involvement Statement

Patients, carers, and members of the public were not involved in the design, conduct, reporting, or dissemination of this research.

## Guidelines and Standards Statement

This study was reported in accordance with the Transparent Reporting of Evaluations with Nonrandomized Designs (TREND) Statement.

## Abbreviations

The following abbreviations are used in this manuscript:

ATP	Adenosine triphosphate
HAIs	Healthcare-Associated Infections
HCA	Healthcare assistant
OR	Operating Room
TREND	Transparent Reporting of Evaluations with Nonrandomized Design
UV	Ultraviolet
